# Impact of age on liver damage, inflammation, and molecular signaling pathways in response to femoral fracture and hemorrhage

**DOI:** 10.3389/fimmu.2023.1239145

**Published:** 2023-08-24

**Authors:** Fanshuai Meng, Yuzhuo Zhou, Alessa Wagner, Jasmin Maria Bülow, Kernt Köhler, Claudia Neunaber, Katrin Bundkirchen, Borna Relja

**Affiliations:** ^1^ Department of Trauma, Hand, Plastic and Reconstructive Surgery, Translational and Experimental Trauma Research, Ulm University Medical Center, Ulm, Germany; ^2^ Department of Trauma and Reconstructive Surgery, Uniklinik RWTH Aachen, Aachen, Germany; ^3^ Department of Trauma Surgery, Hannover Medical School, Hannover, Germany; ^4^ Institute of Veterinary Pathology, Justus Liebig University Giessen, Giessen, Germany

**Keywords:** femur fracture, hemorrhagic shock, aging, liver injury, inflammasome, NF-KappaB, Akt

## Abstract

**Background:**

Trauma causes disability and mortality globally, leading to fractures and hemorrhagic shock. This can trigger an irregular inflammatory response that damages remote organs, including liver. Aging increases the susceptibility to dysregulated immune responses following trauma, raising the risk of organ damage, infections, and higher morbidity and mortality in elderly patients. This study investigates how aging affects liver inflammation and damage post-trauma.

**Methods:**

24 male C57BL/6J mice were randomly divided into four groups. Twelve young (17-26 weeks) and 12 aged (64-72 weeks) mice were included. Mice further underwent either hemorrhagic shock (trauma/hemorrhage, TH), and femoral fracture (osteotomy) with external fixation (Fx) (THFx, n=6) or sham procedures (n=6). After 24 hours, mice were sacrificed. Liver injury and apoptosis were evaluated using hematoxylin-eosin staining and activated caspase-3 immunostaining. CXCL1 and infiltrating polymorphonuclear leukocytes (PMNL) in the liver were assessed by immunostaining, and concentrations of CXCL1, TNF, IL-1β, and IL-10 in the liver tissue were determined by ELISA. Gene expression of *Tnf*, *Cxcl1*, *Il-1β*, and *Cxcl2* in the liver tissue was determined by qRT-PCR. Finally, western blot was used to determine protein expression levels of IκBα, Akt, and their phosphorylated forms.

**Results:**

THFx caused liver damage and increased presence of active caspase-3-positive cells compared to the corresponding sham group. THFx aged group had more severe liver injury than the young group. CXCL1 and PMNL levels were significantly higher in both aged groups, and THFx caused a greater increase in CXCL and PMNL levels in aged compared to the young group. Pro-inflammatory TNF and IL-1β levels were elevated in aged groups, further intensified by THFx. Anti-inflammatory IL-10 levels were lower in aged groups. *Tnf* and *Cxcl1* gene expression was enhanced in the aged sham group. Phosphorylation ratio of IκBα was significantly increased in the aged sham group versus young sham group. THFx-induced IκBα phosphorylation in the young group was significantly reduced in the aged THFx group. Akt phosphorylation was significantly reduced in the THFx aged group compared to the THFx young group.

**Conclusion:**

The findings indicate that aging may lead to increased vulnerability to liver injury and inflammation following trauma due to dysregulated immune responses.

## 1 Introduction

Traumatic injuries account for 8% of all deaths worldwide and account as one of the leading causes of morbidity and mortality worldwide (1). Fatal initial injuries resulting from trauma can cause early death, while secondary infections and resulting complications can lead to (multiple) organ failure and contribute to post-injury mortality (2). Recovery and outcome after major trauma are therefore influenced by post-injury complications (3). Femoral fractures are frequently accompanied with a massive blood loss, both of which are common in patients with multiple trauma, and result in high mortality rates (4, 5).

Cell death from initial tissue damage, blood loss and subsequent secondary tissue damage cause the release of damage-associated molecular patterns (DAMPs) such as adenosine triphosphate (ATP) (6), which contribute to the inflammatory response, as shown e.g. in ischemia/reperfusion (I/R) liver injury triggered by the activation of inflammasomes in Kupffer cells, which contribute to the local inflammatory response (7). Inflammasomes play a critical role in the maturation of Interleukin-1β (IL-1β) and can cause liver injury in I/R due to the release of pro-inflammatory cytokines and excessive neutrophil infiltration (7, 8). Polymorphonuclear neutrophils are the first inflammatory cells to appear at the site of injury, which can also release local pro-inflammatory mediators, such as IL-1β, tumor necrosis factor (TNF) and diverse DAMPs (9–11), activating the nuclear factor kappa-light-chain-enhancer of activated B-cells (NF-κB) pathway. NF-κB regulates inflammation by increasing the production of inflammatory cytokines, chemokines, and adhesion molecules, as well as regulating cell proliferation, apoptosis, and differentiation (12). Activation of the canonical NF-κB pathway occurs through the phosphorylation of the NF-κB inhibitor alpha (IκBα), which is induced in a controlled manner. The level of phosphorylation of IκBα serves as an indicator of NF-κB pathway activation (13, 14). Previous research has shown that NF-κB is also involved in stimulating the activity of protein kinases B (Akt) (15). Akt, a protein involved in cell survival and growth, also plays an important role in inflammation progression (16, 17). Akt activates the IκB kinase IKK, leading to the degradation of IκB and nuclear translocation of NF-κB, which promotes the expression of cytokines, including IL-1β and TNF-α, and accelerates the inflammatory response (18, 19).

Aging, as defined in biology, refers to the gradual increase in frailty of an organism over time, leading to a decreased ability to handle stress (20). In older individuals, there is a commonly observed condition known as “inflammaging”, characterized by a baseline level of inflammation (20–22). Furthermore, aging is associated with a decline in both innate and adaptive immune pathways, resulting in reduced ability to respond to pathogens, a condition known as immunosenescence (21–23). At the cellular level, aging is marked by a chronic, sterile, low-grade inflammation, progressive decline in the function of cells, including their ability to proliferate, differentiate, and carry out physiological functions (21, 22, 24, 25). When injury occurs, the body’s systemic inflammatory response to trauma, may be exaggerated in older people due to higher levels of cytokines or reduced production of anti-inflammatory cytokines (26). The presence of noticeable indications of liver degeneration, hepatic inflammation, and fibrosis is closely linked to old age in mice (27). 24 hours after burn injury, indicators of liver damage and levels of C-X-C Motif Chemokine Ligand (CXCL)1 were increased in aged mice when they were treated with lipopolysaccharide compared to young mice, while IL-6 aged knockout mice exhibited reduced liver injury (28). Similarly, aged mice subjected to ischemia, have shown more activated liver-recruited neutrophils, reduced expression of the cytoprotective heat shock protein 70 in the liver, suppressed and delayed NF-κB activation in response to TNF, all together results indicating that multiple cellular and molecular changes contribute to increased liver injury after ischemia in aged mice (29). Interestingly, also age-dependent NOD-like receptor family, pyrin domain containing (NLRP)3 inflammasome activation, and thus inflammation, impaired the capacity to resolve fibrosis during aging (30).

As society continues to age, the number of elderly individuals experiencing severe trauma, particularly fractures accompanied by blood loss, is expected to rise. The aging process affects both the innate and adaptive immune systems, potentially leading to a diminished immunological response to traumatic events compared to younger individuals. Nevertheless, the precise mechanisms that drive age-related changes in inflammation following trauma are not yet fully understood. Against this backdrop, the objective of this study was to explore the impact of aging on inflammatory alterations and the underlying processes responsible for liver injury in mice subjected to femur fracture and hemorrhagic shock. The study specifically focused on investigating the NF-κB signaling pathway, AKT and inflammasome activation. Our hypothesis suggests that aging may contribute to a heightened inflammatory response and more severe liver damage in the elderly group through trauma-induced inflammasome activation and disrupted NF-κB AKT activation.

## 2 Materials and methods

### 2.1 Animal husbandry

This research obtained approval from the local institutional animal care and research advisory committee and received authorization from the local government of Lower Saxony, Germany (approval number: 33.12-42502-04-17/2491). The study utilized young (17-26 weeks) and aged (64-72 weeks) male C57BL/6J mice obtained from Janvier Labs, Le Genest-Saint-Isle, France (31). The mice were housed individually in cages under standardized conditions at the Central Animal Laboratory of Hannover Medical School. The cages, bedding, and drinking bottles were regularly replaced, and standard softwood granules from Altromin GmbH (Lage, Germany) were used as litter material for the experimental animals.

### 2.2 Group distribution

A total of twenty-four animals were randomly allocated to four groups. The sham groups consisted of six animals in each group (sham young and sham aged). In these groups, the animals underwent femoral artery catheterization at the left leg and were fitted with an external fixator on the right side, but no blood loss or femoral osteotomy was performed. The trauma groups also consisted of six animals in each group (THFx young and THFx aged). In these groups, the animals experienced hemorrhagic shock through blood withdrawal via the left femoral artery catheter and implantation of an external fixator, followed by femoral osteotomy of the right leg. The animals were sacrificed 24 hours after the experimental procedures were conducted.

### 2.3 Experimental model

All surgical procedures were carried out under deep inhalation anesthesia using isoflurane (Baxter Deutschland GmbH, Unterschleißheim, Germany), following the previously described methods (32, 33). The experiment started once the negative interphalangeal reflex in mice was consistently absent. To maintain the mice`s body temperature, a heating pad was used during surgical procedure, and Bepanthen eye ointment was applied to prevent eye dryness. Subcutaneous injections of 5 mg/kg body weight carprofen and 1 mg/kg body weight butorphanol were administered for intraoperative analgesia. Local anesthesia at the surgical site was achieved with prilocaine hydrochloride. Postoperative analgesia was provided by mixing metamizole at a dose of 200 mg/kg body weight into drinking water, and additional subcutaneous injections of carprofen and butorphanol were administered as required. After surgery, the animals were placed in a warm environment under red light until they regained full consciousness, and then housed separately to prevent aggression that could hinder the healing of the surgical wound. The animals’ vital signs and mobility, as well as signs for lameness were regularly monitored and assessed post-surgically.

In both the sham and trauma groups, a catheter was inserted into the left femoral artery and an external fixator (MouseExFix simple L 100%, RISystem, Davos, Switzerland) was implanted in the right femur. Animals in the trauma groups (THFx young; THFx aged) underwent pressure-controlled hemorrhagic shock. For this, blood was withdrawn until the mean arterial blood pressure reached 35 ± 5 mm Hg. The hypovolemic shock state was maintained for a total of 90 minutes. Following that, the animals were re-infused with four times the amount of blood withdrawn (up to a maximum of 2.4 ml) using warm Ringer’s solution within 30 minutes, and the catheter was then removed. The fixator was inserted into the right femoral shaft, and the diaphysis was osteotomized centrally between the two middle pins using a 0.44 mm diameter wire saw (Gigly wire saw, RISystem) in the THFx groups. All wounds were sutured using Prolene 6-0 (Ethicon, Cincinnati, USA), and the animals were allowed to move freely immediately after the completion of the surgical procedures.

### 2.4 Harvesting procedures

After twenty-four hours following the surgery, the animals were euthanized by intraperitoneal injection of 75 mg/kg body weight of ketamine and 1 mg/kg body weight of medetomidine. Once the absence of negative interdigital reflexes was confirmed, the abdominal cavity was opened, and a heparinized sharp 25-gauge syringe was used to puncture the heart and collect blood. Following that, cervical dislocation was performed. The blood was centrifuged at 7000 rpm for 5 minutes at room temperature, and was plasma stored at -80°C until further use. Subsequently, the incision was extended along the chest wall. Twenty ml of PBS was perfused through the heart using a 21-gauge blunt-tipped syringe (BD, Franklin Lakes, USA). The left lateral liver lobe was ligated, swiftly frozen in liquid nitrogen, and stored at - 80°C. Then, for subsequent (immune)histological analyses, mice were perfused with 10 ml of 4% buffered Zn-Formalin (Thermo Fisher Scientific, Waltham, USA) through the heart, and the right liver lobe was removed and fixed overnight for further histological analysis.

### 2.5 Examination of liver damage

The collected specimens were fixed in 4% buffered Zn-formalin (Thermo Fisher Scientific, Waltham, USA) overnight and subsequently embedded in paraffin. The specimens were then sectioned into 3 µm slices to prepare them for hematoxylin-eosin (HE) and immune histological stainings. The paraffin was removed, and the liver sections were rehydrated. Subsequently, the sections were stained with hemalum solution (Carl Roth, Karlsruhe, Germany) for 10 minutes at room temperature. After rinsing in water for 10 minutes, the tissue was counterstained with eosin (Carl Roth, Karlsruhe, Germany) for 3 minutes. The sections were then dehydrated using an ascending alcohol series and mounted with a xylene-based mounting medium (Mountex, Medite Medical GmbH, Burgdorf, Germany). The histological assessment of liver damage in the HE-stained sections from different experimental groups was performed by an independent examiner. Briefly, random necrosis, single cell degeneration/necrosis or individualization, zonal necrosis (perivenous), vacuolization of Ito cells, and vacuolization of hepatocytes were scored individually with 0 (not observed), 1 (mild), 2 (moderate) or 3 (marked). Then, the mean out of all individual values was calculated for each sample to provide the liver injury score (LIS). Furthermore, plasma levels of liver damage marker glutamic oxaloacetic transaminase (GOT) or aspartate aminotransferase were determined by Arkray Spotchem EZ SP-4430.

### 2.6 RNA extraction, reverse transcription and semi-quantitative polymerase chain reaction

RNA extraction was conducted from the liver tissue using the Precellys 24 Homogenizer (Bertin Technologies, Montigny-le-Bretonneux, France) through mechanical disruption. The RNeasy assay buffer (Qiagen, Hilden, Germany) was utilized following the manufacturer’s protocol. To eliminate any remaining DNA, the sample underwent treatment with the RNase-free DNase kit (Qiagen, Hilden, Germany). The RNA was qualitatively and quantitatively analyzed using Tecan’s NanoQuant Plate on the Spark M10 Microplate Reader (Tecan, Männedorf, Switzerland). For cDNA synthesis, the iScript™ cDNA Synthesis Kit (BioRad, Hercules, USA) was employed according to the manufacturer’s instructions. Gene expression levels of *Tnf* (qMnuCED0004141), *Cxcl1* (qMnuCED0047655), *Il-1β* (qMnuCED0045755), and *Cxcl2* (qMmuCED0050757) were quantified using the PrimePCR SYBR Green Assay (BioRad, Hercules, USA) with specific primer sets for mice. The housekeeping gene *Gapdh* (qMnuCED0027467) was quantified as a control. The PCR reaction was performed in a total volume of 25 µl, including the SYBR Green qPCR Master Mix (BioRad), following the manufacturer’s instructions. The reaction took place using the C1000 Touch Thermal Cycler with the CFX96 Touch Real-Time PCR Detection System (BioRad, Hercules, USA). Finally, the relative expression level of each target gene was determined using the comparative threshold-cycle (CT) method (2-ΔΔCT method), which involved normalizing the expression of each target gene to that of *Gapdh*.

### 2.7 Quantification of protein expression levels via ELISA

The liver tissue was homogenized in lysis buffer (FNN0021, Invitrogen™) at 4°C, followed by centrifugation at 20,000 x g for 30 minutes at 4°C. The resulting supernatants were stored at -80°C for future analysis. The protein supernatant extracted from liver tissue was stored at -80°C for later analysis of protein concentration of TNF, IL-1β, IL-10 and CXCL1 using mouse-specific ELISA kits (R&D Systems, Minneapolis, USA) according to manufacturer’s guideline. For measuring of CXCL1 plasma samples were diluted in a ratio of 1:2. ELISA quantification was performed using the Infinite M200 microplate reader (Tecan, Männedorf, Switzerland).

### 2.8 Immunohistological staining of CXCL1, neutrophile elastase and active caspase-3

Paraffin-embedded liver tissue sections (3 µm) were deparaffinized twice for 5 minutes using Roti Histol (Carl Roth, Karlsruhe, Germany), and rehydrated using a gradually decreasing concentration of alcohol at 100%, 90%, and 70%. R-Universal epitope recovery buffer (Aptum, Kassel, Germany) was used to achieve heat-induced epitope retrieval with the 2100-Retriever (Prestige Medical, Blackburn, England) at 121°C for 20 minutes following the manufacturer’s manual. Block of endogenous peroxidase was performed using hydrogen peroxide (Peroxidase UltraVision Block, Thermo Fisher Scientific, Waltham, USA) for 20 minutes. Primary antibodies for CXCL1 (abcam, USA, ab269939, rabbit anti-mouse, 1:300), neutrophile elastase (Bioss, USA, bs-6982R, rabbit anti-mouse, 1:200) or active caspase-3 (Cell Signaling Technology, USA, anti-cleaved caspase-3 (Asp175), #9661, rabbit anti-mouse, 1:300) were diluted according to manufacturer’s recommendation in Antibody Dilution Buffer (Dako Cytomation) and incubated for one hour at room temperature. Subsequently, the secondary antibody conjugated with horseradish peroxidase (Histofine Simple Stain Mouse MAX PO (R), Nichirei Biosciences Inc, 414311F) was incubated for 30 minutes at room temperature and 3-amino-9-ethylcarbazol (AEC, DCS Innovative Diagnostik-Systeme, Hamburg) was used to detect specific binding. Slides were counterstained with hematoxylin (Carl Roth, Karlsruhe, Germany) and mounted (Medite Medical GmbH, Burgdorf, Germany). Imaging was performed using the Zeiss Axio Observer Z1 microscope (40x objective, Zeiss, Göttingen, Germany). The evaluation was performed using ImageJ software. For CXCL1, mean intensity values were measured, while for neutrophile elastase and active caspase-3, positive-counted cells were measured in 25 high-power fields in the 400x magnification.

### 2.9 Western blotting

The liver tissue was homogenized in lysis buffer (FNN0021, Invitrogen™) at 4°C, followed by centrifugation at 20,000 x g for 30 minutes at 4°C. The resulting supernatants were stored at -80°C for future analysis. Electrophoresis was conducted on 15 µg of protein lysate, which was separated by a 10% polyacrylamide sodium dodecyl sulfate gel and subsequently transferred to a polyvinylidene difluoride membrane (Thermo Fisher Scientific). The blots were blocked in a blocking buffer (5% nonfat dry milk in 1 mM Tris, 150 mM NaCl, pH 7.4) for one hour at room temperature. Subsequently, primary antibodies (in 0.5% bovine serum albumin and 0.5% Tween 20) were incubated overnight at 4°C and constant shaking. IκBα Rabbit polyclonal antibody (9242S, 1:1000), Phospho-IκBα Mouse mAb (9246S, 1:1000), Akt Rabbit mAb (4691S, 1:1000), and Phospho-Akt Rabbit mAb (4060S, 1: 2000, all Cell Signaling Technology) were used as primary antibodies. Monoclonal beta-Actin antibody (4967S, 1:2000 Cell Signaling Technology) was used as housekeeping gene. Then, a horseradish peroxidase-conjugated secondary antibody (abcam) was subsequently applied for 1 hour at room temperature on the shaker. For detection of Phospho-Akt, Akt and IκBα Goat Anti-Rabbit IgG H&L (HRP) (ab288151, 1:10000, Abcam) and for Phospho-IκBα Goat Anti-Mouse IgG H&L (HRP) (ab97023, 1:10000, Abcam) was used as secondary antibody. Proteins were detected using 1 ml ECL™ Western blot detection reagent. After measuring phosphorylated IκBα and phosphorylated Akt the PDVF membrane was placed in TBS, and washed 2x15 minutes in stripping buffer (0.2 M Glycin, 0.1% SDS, 1% Tween20, pH 2.2), and 3x5 minutes in TBST on a rocker with 50 rpm. The membranes were then blocked again for one hour in blocking buffer and incubated with IκBα and Akt primary antibodies at 4°C. After incubation, the membranes were washed 2x15 minutes with TBST and 1x15 minutes with TBS (20 mM Tris-Base, 0.15 M NaCl, pH 7.6), and incubated with the corresponding secondary antibody for one hour at room temperature on the shaker. After incubation, the membranes were washed 2x15 minutes with TBST and 1x15 minutes with TBS, and then proteins were detected using 1 ml ECL™ Western blot detection reagent. Then stripping was performed and proceeded to incubate β-actin as described above. At last, the integrated density of individual bands was determined using ImageJ software for quantification of protein level normalization to β-actin by densitometry.

### 2.10 Statistical analysis

The statistical analysis was performed using GraphPad Prism 6 (GraphPad Software, Inc., San Diego, CA). Data were tested for normal distribution using Shapiro-Wilk normality test. As the data was not normally distributed Mann-Whitney U test was performed. The results are presented using box and whiskers and min. to max., and statistically significant differences are indicated as *p<0.05* (*).

## 3 Results

### 3.1 Impact of age on liver damage after THFx

**Figure 1:**
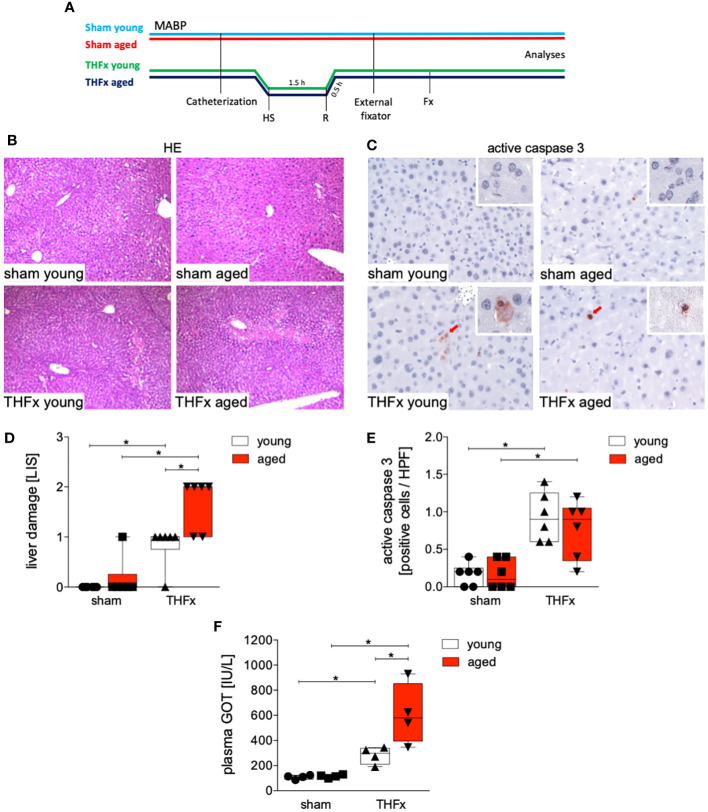
Impact of aging on liver damage after hemorrhagic shock (trauma/hemorrhage, TH) and femoral fracture (Fx). **(A)** The experimental design involved young (17-26 weeks) and aged (64-72 weeks) male C57BL/6J mice, as well as sham and trauma groups, with trauma groups undergoing pressure-controlled hemorrhagic shock (HS) followed by resuscitation (R) with Ringer’s solution and Fx (via osteotomy) (THFx) (green and blue line), while sham groups received catheterization and an external fixator but no THFx induction (light blue and red line). Liver tissue samples were obtained 24 hours after the experiment. MABP: mean arterial blood pressure. **(B)** Representative liver sections upon hematoxylin/eosin (HE) staining, and **(C)** representative liver sections upon the staining of active caspase-3 of the sham young, sham aged, THFx young and THFx aged groups. Red cells are active caspase-3 positive stained cells. **(D)** Quantification of liver damage. **(E)** Quantification of active caspase-3 positively stained cells per high power field (HPF) **(D, E)**; n=6 in each group. **(F)** Quantification of plasma glutamic oxaloacetic transaminase (GOT); n=4 in each group. * p<0.05 between indicated groups.

The experimental design is shown in **Figure 1A**. Hepatic damage resulting from THFx was evaluated by analyzing liver tissue using HE staining (**Figure 1B**), and a comparison was made among the groups (**Figure 1D**). The THFx young group exhibited a significant increase in liver damage compared to the sham young group (p<0.05, **Figure 1D**). Similarly, the THFx aged group displayed a significant increase in liver necrosis compared to the sham aged group (p<0.05, **Figure 1D**). Moreover, there was a significant elevation in liver damage in the THFx aged group compared to the THFx young group (p<0.05, **Figure 1D**).

**Figure 1C** presents the apoptosis induction by THFx in the liver, as demonstrated by immunohistochemical staining of activated caspase-3 (red cells, **Figure 1C**). The quantification of cells positive for active caspase-3, serving as a direct indicator of apoptosis, is presented in **Figure 1E**. In the THFx young group, a significant increase in caspase-3 positive stained cells compared to the sham young group was observed (p<0.05, **Figure 1E**). Additionally, the THFx aged group exhibited a significant increase in caspase-3 positive stained cells compared to the sham aged group (p<0.05, **Figure 1E**). Plasma GOT levels after THFx compared to the respective sham group were significantly increased (p<0.05, **Figure 1F**). Moreover, there was a significant increase in GOT levels in the THFx aged group compared to the THFx young group (p<0.05, **Figure 1F**).

### 3.2 Impact of age on CXCL1 protein expression, systemic CXCL1 levels and neutrophilic infiltration in the liver after THFx

**Figure 2:**
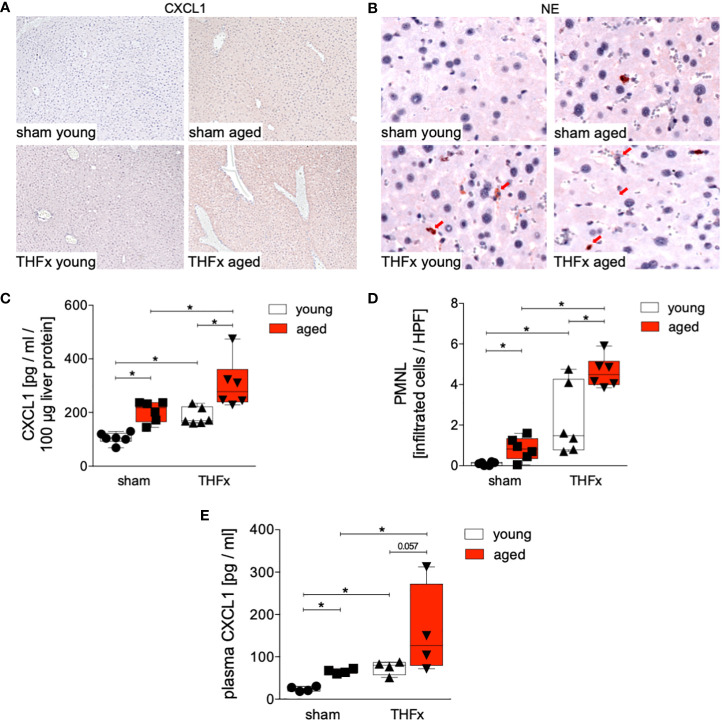
Impact of aging on CXCL1 protein expression and neutrophilic infiltration after hemorrhagic shock (trauma/hemorrhage, TH) and femoral fracture (Fx). The experimental design involved young (17-26 weeks) and aged (64-72 weeks) male C57BL/6J mice, as well as sham and trauma groups, with trauma groups undergoing pressure-controlled hemorrhagic shock followed by resuscitation (R) with Ringer’s solution and Fx (via osteotomy) (THFx), while sham groups received catheterization and an external fixator but no THFx induction. Liver tissue samples were obtained 24 hours after the experiment. **(A)** Representative liver sections upon the staining of CXCL1 in the sham young, sham aged, THFx young and THFx aged groups. **(B)** Representative immunohistological staining of neutrophil elastase (NE) as a marker of polymorphonuclear leukocytes (PMNL) in liver sections. Red arrows indicate NE-positive stained cells. **(C)** Quantification of CXCL1 protein concentration using mouse-specific ELISA-kits in liver. **(D)** Quantification of NE-positive stained cells per high power field (HPF); n=6 in each group, **(E)** Quantification of plasma CXCL1 levels; n=4 in each group. *: p<0.05 between indicated groups.

**Figure 2A** depicts representative liver sections following immunohistochemical staining of CXCL1, while **Figure 2C** illustrates the protein expression of CXCL1 for each group. The liver exhibited a significant increase in CXCL1 protein expression after THFx in both young and aged animals compared to the corresponding sham groups (p<0.05, **Figure 2C**). Moreover, CXCL1 protein expression was significantly higher in the aged groups compared to the respective young groups, particularly in the THFx aged group (p<0.05, **Figure 2C**).

Given that CXCL1 acts as a chemoattractant for various immune cells, notably neutrophils, we investigated the recruitment of polymorphonuclear leukocytes to the liver after THFx. PMNL infiltration in liver tissue was assessed through immunohistochemical staining (**Figure 2B**), and the quantification of positively stained cells (red cells) is presented in **Figure 2D**. There was a significant increase in PMNL infiltration in the THFx young group compared to the sham young group (p<0.05, **Figure 2D**). Similarly, the THFx aged group exhibited a significant increase in PMNL infiltration compared to the sham aged group (p<0.05, **Figure 2D**). Additionally, the aged groups displayed a significant increase in PMNL infiltration compared to the respective young group (p<0.05, **Figure 2D**). Plasma CXCL1 levels after THFx compared to the respective sham group were significantly increased (p<0.05, **Figure 2E**). Moreover, there was an increase in aged groups compared to young groups (p<0.05, **Figure 2E**).

### 3.3 Impact of age on liver inflammatory cytokines after THFx

**Figure 3:**
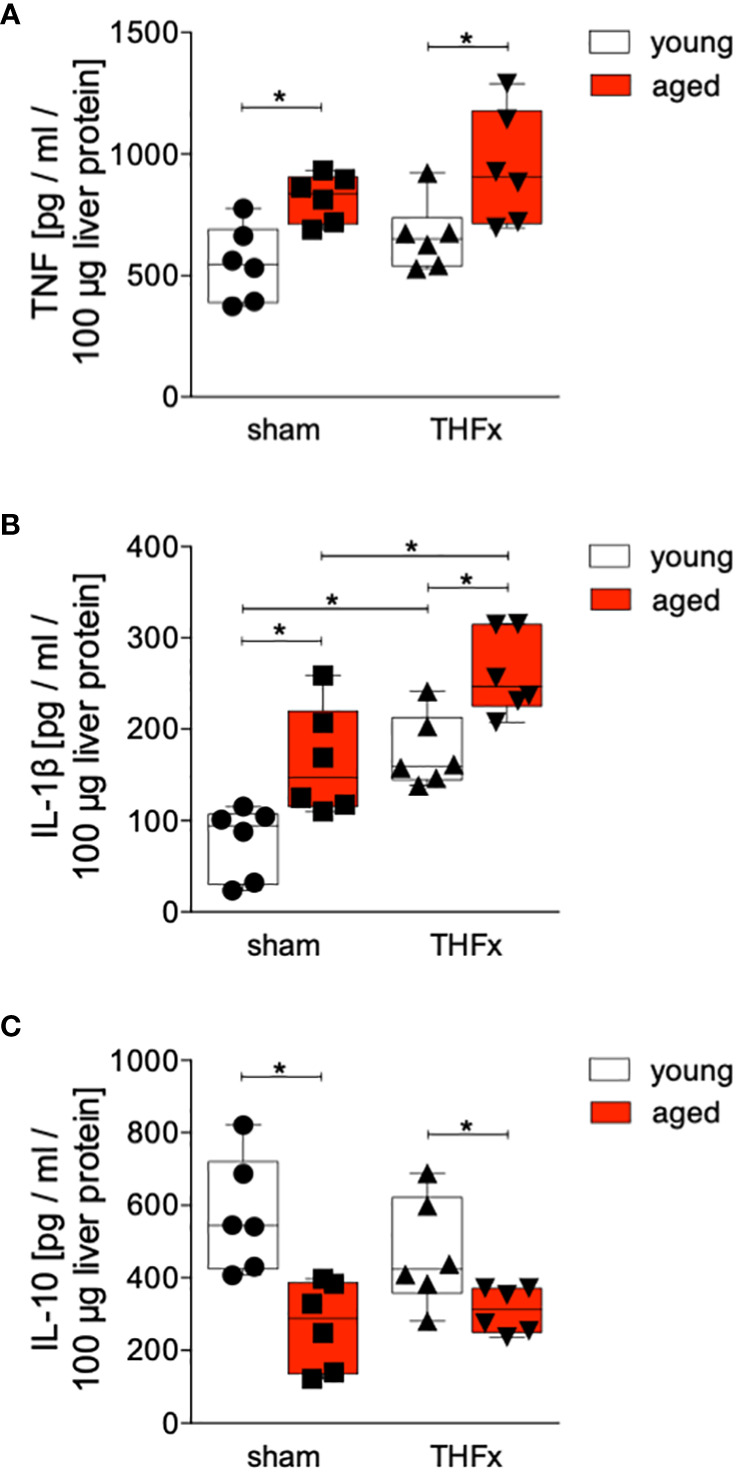
The impact of aging on TNF, IL-1β and IL-10 protein expression after hemorrhagic shock (trauma/hemorrhage, TH) and femoral fracture (Fx). The experimental design included young (17-26 weeks) and aged (64-72 weeks) C57BL/6J mice in both sham and trauma groups. The trauma groups underwent a pressure-controlled hemorrhagic shock and Fx (via osteotomy) (THFx), while the sham groups underwent catheterization and received an external fixator, but no THFx was induced. After 24 hours, the mice were euthanized, and sampling was performed. Quantification of **(A)** TNF, **(B)** IL-1β as well as **(C)** IL-10 protein concentration in liver tissue using mouse-specific ELISA-kits is shown. n=6 in each group, *: p<0.05 between indicated groups.

Protein levels of TNF, IL-1β and IL-10 in homogenized liver tissue were assessed. The results showed that protein concentration of TNF in liver tissue was significantly increased in both aged groups, sham and THFx, compared to the corresponding young group (p<0.05, **Figure 3A**).

The liver exhibited a significant increase in IL-1β protein level after THFx in both young and aged animals compared to the corresponding sham groups (p<0.05, **Figure 3B**). Moreover, the aged groups showed a significant rise in IL-1β protein concentration compared to the respective young groups (p<0.05, **Figure 3B**), particularly, the THFx aged group displayed a significant elevation in IL-1β compared to the THFx young group (p<0.05, **Figure 3B**).

The concentration of IL-10 in liver tissue was significantly decreased in both aged groups, sham and THFx, compared to the corresponding young group (p<0.05, **Figure 3C**).

### 3.4 Impact of age on *Tnf, Cxcl1, Il-1β*, and *Cxcl2* gene expression after THFx

**Figure 4:**
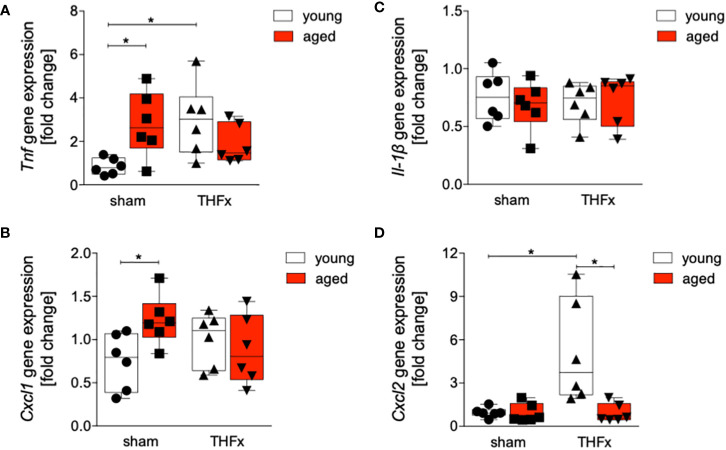
Impact of age on Tnf, Cxcl1, Il-1β and Cxcl2 gene expression after hemorrhagic shock (trauma/hemorrhage, TH) and femoral fracture (Fx). The experimental design included young (17-26 weeks) and aged (64-72 weeks) C57BL/6J mice in both sham and trauma groups. The trauma groups underwent a pressure-controlled hemorrhagic shock and Fx (via osteotomy) (THFx), while the sham groups underwent catheterization and received an external fixator, but no THFx was induced. After twenty-four hours, the mice were euthanized, and sampling was performed. The relative gene expression of **(A)** Tnf, **(B)** Cxcl1, **(C)** Il-1β as well as **(D)** Cxcl2 normalized to Gapdh after qRT-PCR was calculated by using the comparative threshold-cycle 2-ΔΔCT method.; n=6 in each group, *: p<0.05 between indicated groups.

**Figures 4A-D** shows the results of *Tnf*, *Il-1β*, *Cxcl1* and *Cxcl2* gene expression. The relative gene expression of *Tnf* in liver tissue was significantly higher in THFx young group compared to the sham young group (p<0.05, **Figure 4A**). Baseline *Tnf* gene expression in the aged sham group was significantly higher compared to the corresponding young group (p<0.05, **Figure 4A**).

The relative gene expression of *Cxcl1* was significantly increased in the sham aged group compared to the sham young group (p<0.05, **Figure 4B**). There were no significant differences in *Il-1β* gene expression among all examined groups (**Figure 4C**). A significant upregulation of relative gene expression of *Cxcl2 in the* THFx young group compared to all other relevant groups was detected (p<0.05, **Figure 4D**).

### 3.5 Impact of age on Akt and IκBα activation after THFx

**Figure 5:**
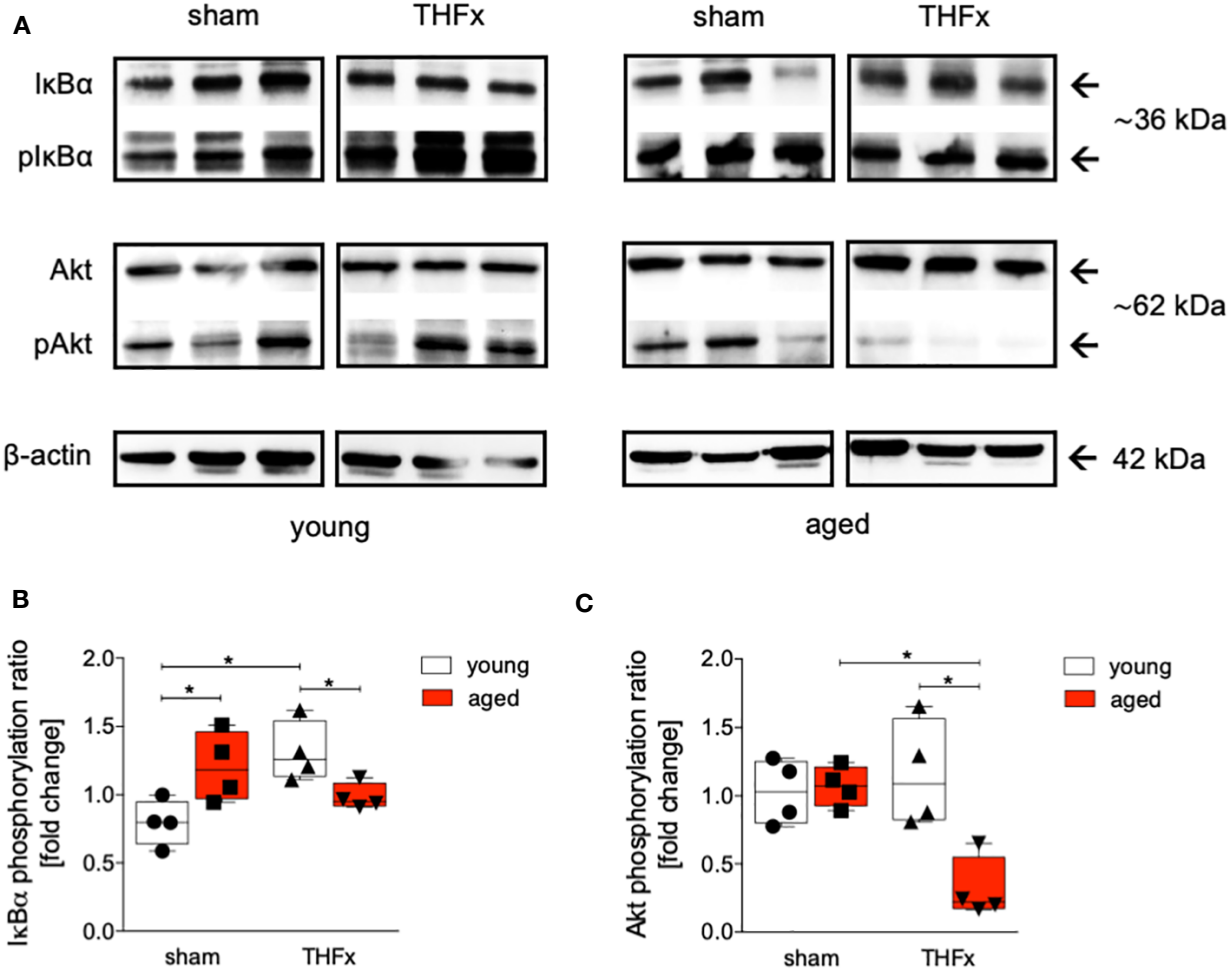
The effect of aging on IκBα and Akt following hemorrhagic shock (trauma/hemorrhage, TH) and femoral fracture (Fx) in young (17-26 weeks) and aged (64-72 weeks) male C57BL/6J mice. The experimental design included young (17-26 weeks) and aged (64-72 weeks) C57BL/6J mice in both sham and trauma groups. The trauma groups underwent a pressure-controlled hemorrhagic shock and Fx (via osteotomy) (THFx), while the sham groups underwent catheterization and received an external fixator, but no THFx was induced. After twenty-four hours, the mice were euthanized, and sampling was performed. **(A)** Western blot analysis of phosphorylated and non-phosphorylated IκBα, AKT as well as β-Actin, and quantification of **(B)** the phosphorylation ratio of IκBα, and **(C)** AKT in liver tissue. n=4 in each group, *: p<0.05 between indicated groups.

The impact of aging on Akt and NF-κB pathways was investigated after THFx. Liver tissue homogenates were collected 24 hours after resuscitation, and western blot analysis was performed to measure phosphorylated and non-phosphorylated IκB and Akt. A significant increase in the IκBα phosphorylation ratio in the sham aged group compared to the sham young group was observed (p<0.05, **Figures 5A, B**). Additionally, the THFx young group exhibited a significant increase in IκB phosphorylation ratio compared to the sham young group (p<0.05, **Figure 5B**). The THFx aged group displayed a significant decrease in the IκBα phosphorylation ratio compared to the THFx young group as well as to the sham aged group (p<0.05, **Figure 5B**).

The phosphorylation ratio of Akt was significantly decreased in the THFx aged group compared to all other relevant groups (p<0.05, **Figures 5A, C**).

## 4 Discussion

Trauma is a major cause of illness and death worldwide, with initial injuries leading to early mortality and complications contributing to organ failure and post-injury mortality (2, 3). Inflammation plays a crucial role in the recovery and outcome after trauma. Additionally, aging affects the immune system and leads to a chronic, low-grade inflammation known as “inflammaging” (20). Older individuals may have an exaggerated inflammatory response to trauma due to higher cytokine levels or reduced production of anti-inflammatory cytokines (23). In mice, aging is associated with liver degeneration, inflammation, and impaired resolution of fibrosis (27). As the population ages, the number of elderly individuals experiencing severe trauma is expected to rise. Therefore, a deeper understanding of the mechanisms behind age-related changes in inflammation following trauma is important. Our study aimed to investigate the impact of aging on inflammatory alterations and liver injury in mice with femur fracture and hemorrhagic shock. Specifically, it was investigated if the NF-κB as well as Akt signaling pathways, and inflammasome activation are involved in aging-induced heightened inflammatory response and more severe liver damage in mice.

The findings indicate that aging enhances vulnerability to liver injury and inflammation following trauma. In the aged group, more severe liver damage was observed (**Figure 1**), along with elevated levels of the pro-inflammatory chemokine CXCL1 and PMNL infiltration in the liver (**Figures 2A–D**). Additionally, the pro-inflammatory cytokines TNF and IL-1β were higher in the aged groups (**Figures 3A, B**), while the anti-inflammatory cytokine IL-10 was lower (**Figure 3C**). Gene expression analysis revealed upregulated *Tnf* and *Cxcl1* in the aged sham group (**Figures 4A, B**). However, no significant differences in gene expression were observed between the young and aged trauma/hemorrhage groups, except for *Cxcl2* (**Figure 4C**), suggesting a greater impact of aging on protein rather than gene expression levels. Moreover, signaling pathways related to inflammation regulation, such as IκBα phosphorylation and AKT phosphorylation, were impaired in the aged trauma/hemorrhage group (**Figures 5**). In conclusion, aging amplifies liver inflammation and damage following trauma, as evidenced by the aged group’s heightened susceptibility, elevated inflammatory markers, immune cell presence, and disrupted signaling pathways.

The liver plays a crucial role in mediating inflammation by producing acute-phase proteins and modulating immune cell activity (34). It is involved in regulating systemic inflammation and secreting pro-inflammatory cytokines (35). Aging is associated with chronic, low-grade inflammation, which negatively affects physiological function (24). In the context of trauma, the liver’s function in regulating inflammation becomes even more significant. Severe trauma triggers an inflammatory response involving various immune components including among others inflammatory cytokines, and PMNL activation, that can lead to liver injury and multiple organ dysfunction as well as increased mortality after trauma (26, 36, 37). In line with these reports, our results demonstrate increased production and release of pro-inflammatory cytokines and chemokines, IL-1β, TNF and CXCL1 in the liver after THFx (**Figures 2C**, **3A, B**). Similarly, in a fracture-based trauma hemorrhagic liver injury model in rats, it was shown that IL-1β, TNF-α, and CXCL2 increased significantly post-trauma, indicating that inflammatory reactions had occurred in the fraction-induced hemorrhage of liver (38). As also evidenced in our study and as reported before, the activity of the immune system as for example displayed by the increased numbers of infiltrating PMNL in the liver post-trauma (**Figure 2D**), indicates that injury-induced inflammation stimulates immune cells, and that these changes are associated with aggravations of liver injury (38). Recently, it was shown that apoptosis biomarkers including activated and cleaved caspase-3 were increased in a long-term large animal polytrauma model, as also confirmed by our results (**Figure 1E**), supporting the damage of the liver after trauma (39, 40). Furthermore, our results revealed a significant activation of the inflammasome by increased IL-1β levels in mice with THFx (**Figure 3B**), which is consistent with findings of Sadatomo et al. showing that liver injury in ischemia/reperfusion arised from pro-inflammatory cytokines and excessive neutrophil infiltration was inflammasome-mediated (7, 8). The observed changes involve the modulation of signaling pathways, particularly those associated with NF-κB activation. Activation of NF-κB triggers the production of pro-inflammatory mediators such as cytokines, chemokines, and enzymes, which in turn, stimulate further NF-κB activation and its upstream signaling machinery, creating a positive feedback loop that intensifies the inflammatory response mediated by NF-κB and tissue injury after trauma (41–44). Enhanced activation of IκBα after THFx suggests that NF-κB activation contributes to the THFx induced increase of the pro-inflammatory effects related to increased cytokine and chemokine production, inflammasome activation and ensuing liver inflammation and injury.

In the field of trauma and inflammation research, most studies have traditionally focused on very young animals, typically between 6 to 12 weeks of age. While these studies have provided valuable insights into the mechanisms of disease, they may not accurately reflect the processes occurring in adult or aged humans. To address this limitation, we conducted a direct comparison between the response to THFx injury in “young/mature adult” mice (17-26 weeks old) and “aged adult” mice (64-72 weeks old). Our aim was to identify the differences between these two age groups and understand how they relate to humans of different age ranges. According to life tables, mice aged 17-26 weeks correspond to approximate human age of approximately above 18 years of age years, while mice aged 64-72 weeks correspond to approximate human age of above 60 years of age (45). Aging or senescent cells and/or tissues secrete a variety of bioactive molecules, including pro-inflammatory cytokines such as IL-1β, chemokines, growth factors, and proteases, collectively referred as senescence-associated secretory phenotype (SASP), that describe a chronic low-grade inflammation, also known as inflammaging (21, 46). This is in line with our findings showing enhanced TNF, IL-1β, and CXCL levels in the liver of aged sham animals (**Figures 2C**, **3A, B**). The retrograde breaching of the endothelium by neutrophils was governed by enhanced production of the chemokine CXCL1 from mast cells that localized at endothelial cell junctions in aged mice (47). Thus, with implication to the dysregulated systemic inflammation associated with aging, the authors conclude that neutrophils stemming from a local inflammatory site contribute to remote organ damage (47). This is in line with our findings showing enhanced neutrophil infiltration into the liver from aged mice (**Figure 2D**). A large array of inflammatory factors involved in ageing is linked to the NF-κB system, and it was shown that NF-κB activation has been implicated in various age-related diseases and conditions, including chronic inflammation, neurodegenerative disorders, and certain cancers (48). Our findings underlining IκBα activation in aged sham animals also suggest that the dysregulated inflammatory response leading to the release of pro-inflammatory markers and excessive neutrophil activation was associated with NF-κB signaling pathway as potential ageing phenomenon (**Figure 5B**).

The post-traumatic inflammatory response and the function of NF-κB in aged liver is complex. Clearly, an enhanced vulnerability to inflammation and liver injury following trauma was present. In the aged THFx group, elevated levels of the pro-inflammatory proteins CXCL1, TNF and IL-1β, and reduced levels of the anti-inflammatory cytokine IL-10, compared to the young THFx group were observed (**Figures 2C**, **3A–C**). The local CXCL1 concentrations in the liver were more than doubled compared to systemic CXCL1 levels, indicating, that the hepatic CXCL1 increase is rather induced by a local response than a systemic effect. Along with the elevated hepatic CXCL1 levels, enhanced neutrophil infiltration into the livers of aged animals were observed (**Figures 2C, D**). This is in line with previous findings from an *in vivo* endotoxin model showing elevated hepatic neutrophil numbers and their activity in aged relative to young animals receiving the same endotoxin stimulus (28). Such presence of hepatic inflammation, and liver degeneration, was closely linked to old age in mice (27), while further data indicate that CXCL1 levels and liver damage were higher in aged mice when they were treated with lipopolysaccharide compared to young mice, while e.g. an IL-6 knockout in aged mice exhibited reduced liver injury (28). Although there were noticeable alterations in proteins within aged animals following THFx, the corresponding changes in gene expression levels were not as pronounced. This implies either a disconnect between the observed changes at the protein level and the corresponding genetic regulation, or that the peak expression period of the pro-inflammatory genes has not been captured upon harvesting the livers of the mice 24 hours after THFx. Hill et al. found that gene expression of IL-1β and TNF peaked within 10-18 hours after cerebral ischemia-reperfusion injury in C57BL/6J mice (49). Similarly, Pestka and Amuzie demonstrated that deoxynivalenol-induced gene expression of IL-1β and TNF peaked after one hour in mouse livers (50). To obtain more precise mRNA data for inflammatory factors, future studies should consider adding additional time points for liver harvesting. It is worth noting that gene expression is a dynamic process influenced by various factors. The effects of THFx might primarily affect post-transcriptional or translational processes, impacting protein function or stability without causing significant changes in gene expression. Several factors could contribute to this discrepancy. Additionally, it is important to consider the complexity of gene regulation mechanisms, that can be influenced by a wide range of factors, including transcription factors, epigenetic modifications, and non-coding RNAs. It is possible that THFx primarily affects specific post-transcriptional or translational processes, leading to changes in protein levels without significant alterations in gene expression. This is underlined by the findings of Nyugen et al. and Renshaw et al. regarding the association between aging and impaired Pattern Recognition Receptor (PRR) signaling, particularly focusing on Toll-like receptors (TLRs) in innate immune cells (51, 52). Nyugen et al. demonstrated that aging is linked to impaired signaling through PRRs, possibly due to alterations in TLR protein level in innate immune cells when comparing aged to young individuals (51). Similarly, Renshaw et al. reported significant age-related decreases in TLR gene expression in mice, specifically observing a general decrease in the expression of *Tlr1-Tlr9* genes in C57BL/6 aged mice (52). Since the activation of the NF-κB pathway occurs through TLRs on the cell membrane, the reduced expression of TLRs in aged mice may lead to a decrease in NF-κB pathway activation. Also, it was shown before, that NF-κB activation in the liver is greatly reduced in aged mice compared to young mice after hepatic ischemia/reperfusion injury due to the decreased proteasome subunit, non-ATPase 4 expression preventing recruitment of phosphorylated and ubiquitinylated IκBα to the proteasome, resulting in a defect in NF-κB activation (29, 53). This decrease in NF-κB pathway activation is supported by the reduced IκBα phosphorylation ratio in the aged group compared to the young group in the current study utilizing the THFx model (**Figure 5B**). Also, the crosstalk between the NF-κB and Akt pathway is important in various cellular processes including cell survival, inflammation, and immune response (54, 55). While NF-κB can activate Akt signaling, Akt can also regulate NF-κB activity (54, 55). Since the downregulated AKT phosphorylation was accompanied by reduced IκBα phosphorylation after THFx in aged animals in the current study (**Figures 5B, C**), the overall link between Akt and NF-κB suggests a complex regulatory network where both molecules can influence each other’s activity and downstream effects in THFx. This interplay between Akt and NF-κB potentially orchestrating the cellular responses to THFx and maintaining cellular homeostasis is deregulated in aged animals. However, the increase in IκBα phosphorylation ratio in the sham aged group may also suggest that other inflammatory pathways, mediated by TNF and IL-1β, might be activated. Interestingly, age-dependent inflammasome activation, impairing the capacity to resolve fibrosis during aging as shown before (30), has been observed in our study as well by enhanced IL-1β protein levels in aged livers following THFx (**Figure 3B**). Thus, the findings suggest that aging may contribute to a heightened inflammatory response and more severe liver damage in the elderly group through trauma-induced inflammasome activation and disrupted NF-κB, Akt activation.

This study acknowledges several limitations that should be considered when interpreting the results. Firstly, the study was conducted using only male mice, and therefore the conclusions drawn from the study can only be applied to male subjects. In future studies, it is important to examine the effect of sex on the inflammatory response and liver damage caused by aging to obtain a more comprehensive understanding of the topic. Another limitation is that the mice used in the study were healthy, which may not fully represent the complexity of patients in a clinical setting who often have concomitant diseases and more intricate systemic conditions. Additionally, the experimental model used in this study involved implanting an external fixator before performing the osteotomy, whereas in clinical practice, fractures occur before fracture fixation. This difference in the sequence of events may have affected the experimental results and should be considered when interpreting the findings. Moreover, the fracture model used in the study employed a wire saw to create a regular fracture, while clinical situations involve various types of fractures and complex scenarios. Furthermore, the study induced controlled hemorrhagic shock in the animal model, whereas uncontrolled bleeding occurs in actual trauma situations. Such controlled settings may not fully reflect the complexity and severity of fractures and trauma-related bleeding, which should be considered when extrapolating the results to clinical practice. The study primarily focused on the local inflammatory response and liver damage, but it did not include indicators of systemic inflammatory response and liver damage, such as inflammatory mediators and liver enzymes in the blood. Including these indicators in future studies would provide a more comprehensive understanding of the overall effects. Lastly, different inflammatory response and liver damage indicators may appear at different time points. To account for this, future studies should consider including multiple time points to capture the dynamic changes and simulate the clinical scenario more accurately, thereby obtaining more comprehensive and accurate results.

Overall, the findings demonstrate that aging exacerbates liver inflammation and damage following trauma, supported by increased susceptibility, elevated inflammatory markers, immune cell presence, inflammasome activation, and disrupted signaling pathways in the aged group.

## Data availability statement

The raw data supporting the conclusions of this article will be made available by the authors, without undue reservation.

## Ethics statement

The animal study was approved by local government of Lower Saxony, Germany (approval number: 33.12-42502-04-17/2491). The study was conducted in accordance with the local legislation and institutional requirements.

## Author contributions

Conceptualization, CN and BR. Methodology, FM, YZ, KK, and KB. Validation, FM, KB, and BR. Formal analysis, FM and BR. Investigation, FM, YZ, and KB. Resources, CN and BR. Data curation, FM and BR. Writing-original draft preparation, FM, KB, and BR. Writing-review and editing, AW and JB. Visualization, FM and BR. Supervision, KB, CN, and BR. Funding acquisition, CN and BR. All authors contributed to the article and approved the submitted version.
